# The transfer of knowledge on integrated care among five European regions: a qualitative multi-method study

**DOI:** 10.1186/s12913-019-4865-8

**Published:** 2020-01-03

**Authors:** Liset Grooten, Hubertus Johannes Maria Vrijhoef, Tamara Alhambra-Borrás, Diane Whitehouse, Dirk Devroey

**Affiliations:** 1 Department of Family Medicine and Chronic Care, Vrije Universiteit Brussel, P.O. 103, B-1090, Brussels, Belgium; 2 Department Patient & Care, Maastricht University Medical Center, Maastricht, the Netherlands; 3 Panaxea B.V, Amsterdam, the Netherlands; 4 Department of Family Medicine and Chronic Care, Vrije Universiteit Brussel, Brussel, Belgium; 5 Polibienestar Research Institute, Universitat de Valencia, Valencia, Spain; 6 European Health Telematics Association (EHTEL), Brussels, Belgium

**Keywords:** Knowledge transfer, Delivery of health care, Integrated, Scaling-up

## Abstract

**Background:**

To examine how the knowledge transfer processes unfolded within SCIROCCO, a EU funded project (3rd Health Programme (2014–2020)) that aimed to facilitate the process of knowledge sharing across five European regions, to speed up adoption and scaling-up of integrated care initiatives.

**Methods:**

A qualitative multi-method design was used. Data collection methods included focus groups, project documents and action plans of the regions. The data was analysed using a qualitative content-analysis procedure, which was guided by the frameworks of knowledge exchange and the why, whose, what, how framework for knowledge mobilisers.

**Results:**

All five components (including the themes) of knowledge exchange could be identified in the approach developed on the knowledge transfer processes. The four questions and accompanying categories of the framework of knowledge mobilisation were also identified to a large degree.

**Conclusions:**

The observed incorporation of distinct forms of knowledge from multiple sources and the observed dynamic and fluid knowledge transfer processes both suggest that SCIROCCO developed a comprehensive knowledge transfer approach aiming to enable the adoption and scaling-up of integrated care. Overall, the multi-method qualitative nature of this research has allowed some new and practical insights in the knowledge transfer activities on integrated care between several European regions. To obtain a clear understanding of the content of the knowledge transfer approaches, which could assist the operationalising of models to support the evaluation of knowledge transfer activities, it is strongly recommended that further research of this type should be conducted in other research settings.

## Introduction

An increasing amount of knowledge is obtained on different models and approaches of integrated care implemented across a variety of settings [1]. Many definitions of integrated care exist of which one defines integrated care as “patient-centred, proactive and well-coordinated multidisciplinary care, using new technologies to support patients’ self-management and improve collaboration between caregivers.” [2] Nolte and colleagues, who looked into the experiences of 12 countries in Europe in their efforts to enhance the care for people with chronic conditions, found that innovative care models - that challenge established ways of organising services - are often implemented as time-limited pilot or small-scale, localised projects [3]. To obtain more benefit for people from the advantages seen in promising innovative practices, it is important to learn from and share successful experiences. The spread of insights is believed to support the scaling-up of favourable initiatives and innovations. However, it is implausible that a pilot offers much support for the next implementation: as Pawson points out, the interaction between the intervention and its context determines outcome, meaning that the determinants of success change in another context [4]. The question of how to spread innovative initiatives and, at the same time, do justice to recognising the need, context, culture and resource availability of different settings remains for the most part unanswered.

To encourage knowledge transfer (KT) and scaling-up of innovative integrated care solutions, the SCaling IntegRated Care in COntext (SCIROCCO) project has tested and validated the SCIROCCO tool (‘the tool’), which is argued to support the adoption of integrated care across Europe [5]. Using a step-based approach, the tool was developed and assessed in real life settings within the project. The SCIROCCO project aimed to test an approach to transfer knowledge between five European regions using the tool to assist in this process of information sharing and KT across the regions.

In the literature, transfer of knowledge is known by a diversity of terms, e.g. knowledge transfer and exchange, knowledge translation or utilisation and knowledge mobilisation [6, 7] and mostly considered to be an approach to exchange knowledge between research and practice. The process of KT is suggested to be a dynamic and iterative process taking place among a complex system of interactions. Hence, a frequently cited definition is the one of the Canadian Institutes of Health Research, which describes knowledge translation as “as a dynamic and iterative process that includes synthesis, dissemination, exchange and ethically sound application of knowledge to improve the health of Canadians, provide more effective health services and products and strengthen the health care system. This process takes place within a complex system of interactions between researchers and knowledge users which may vary in intensity, complexity and level of engagement depending on the nature of the research and the findings as well as the needs of the particular knowledge user [8].“.

To ensure a solid and successful process of moving knowledge to practice, several scholars have recommended to guide the KT activity by using a model displaying how the process works and how it can support knowledge producers and users to plan and evaluate the activities [9–12]. Unfortunately, partly because there is such disparity across the field, there are relatively few tools and mechanisms for evaluating knowledge mobilisation projects [13]. Furthermore, there is a lack of sufficiently empirically tested and operationalised models to adequality guide KT activities. Davies et al., found in their “review of reviews” in the area of knowledge mobilisation “a bewildering variety” of models, theories and frameworks in health care, education and social care [14]. Many of the models they investigated “were primarily descriptive of the processes around knowledge creation/flow/application, and tend not to be explicit about the necessary configurations, actions or resources that will underpin successful knowledge mobilisation.” Furthermore, with a few exceptions, the models were found to be subjected to limited empirical testing [14].

The SCIROCCO project tested a unique process of KT and information flow among five European regions using the tool to assist this process. Since the transfer of knowledge is suggested to be a complex process and SCIROCCO’s approach took place between several regions, including different health systems, at the start of the project it was not known how the process would unfold in practice. Despite the lack of adequate models to guide the evaluation of the KT approach, it was considered important to gain insight into the way in which the processes of the KT activities in SCIROCCO would play out.

This study, therefore, has the objective to examine the KT process which was designed and tested within the SCIROCCO project to assess whether the approach is meaningful in light of two existing frameworks on KT. Providing an understanding of the processes involved in the SCIROCCO KT process is valuable, as this is expected to provide insight in how SCIROCCO’s KT process intended to add value to the participating regions. Furthermore, the expected insights can support policymakers and stakeholders in other regions in terms of what issues to consider when they are interested in using the tool and processes in order to achieve KT with other regions and to eventually scale-up integrated care initiatives.

### Conceptual frameworks

#### The framework of knowledge exchange (KE)

To provide insight into the complex process of KT that occurred during the SCIROCCO project, this evaluation study is guided by the framework for KE developed by Ward and colleagues [10]. The authors suggested that the framework can be used to gather evidence from case studies of KE interventions and recommended that it could also be used as a template for evaluating KE activities. The initial conceptual framework on KE was developed out of a review of 28 different models which focused on explaining the KT process. Five common components of the KT process identified were [15]:

- Identifying and communicating about the *problem* which the knowledge needs to address;
- Analysing the *context* which surrounds the producers and users of knowledge;
- Developing and selecting the *knowledge* to be transferred;
- Selecting specific KT activities or *interventions*;
- Considering how the knowledge will be *used* in practice.

Subsequently, the authors empirically tested the framework and refined it. As a result, the five components were found to “all be in play at any one time and do not occur in a set order [10].”

#### The framework of knowledge mobilisation

Effectively sharing knowledge requires different strategies depending on who is sharing the knowledge, what knowledge is being shared, how it is shared, and the purpose for which it is shared [16]. More recently, Ward developed a framework for knowledge mobilisers based on a review of 47 knowledge mobilisation models. The framework consists of four questions: Why is knowledge being mobilised?; Whose knowledge is being mobilised?; What type of knowledge is being mobilised?; How is knowledge being mobilised? [7]. Ward argues that these questions and accompanying categories can help knowledge mobilisers reflect on, communicate, and evaluate their aims and objectives, increasing clarity and understanding [7]. The framework is designed to help those involved in knowledge mobilisation to reflect on their personal and/or project-related aims and objectives in a structured way. Therefore, this framework was also used in this study to examine the KT processes within the SCIROCCO project.

## Methods

A qualitative multi-method study was undertaken as this was regarded to be best suited to obtain a detailed understanding of how the KT processes within the SCIROCCO project unfolded.

### Setting

This study took place within the European funded SCIROCCO project under the 3rd Health Programme (2014–2020). From its start (April 2016) till its end (November 2018) the SCIROCCO project tested a strategy which was designed to explore how available knowledge and experiences on integrated care models can be shared to enable “easier and faster” adaptation and implementation in other settings. Several work packages (WPs) within the project were responsible to implement the project objective. Partners from five participating regions (the Basque Country, Norrbotten, Olomouc, Puglia, Scotland) and two organisations conducted the work in the WPs. This study was conducted by members of the project’s third work package (WP3), who were responsible for evaluation activities within the project. The strategy included the next roughly described steps:

- Step 1: In the project, a total of 15 good practices were selected as viable good practices in integrated care in five participating European. These were selected by means of a viability assessment using a form which was developed during the implementation. Within SCIROCCO good practices were defined as inspiring real-life examples of successfully applied innovations in integrated care. Second, the maturity requirements for transfer of these 15 good practices were assessed using the new developed SCIROCCO tool,^1^ resulting in an overview (radar diagram) of the requirements of the local context in which the practices have been developed.
- Step 2: In the next step, the five regions assessed their maturity in the provision of integrated by using the SCIROCCO tool. The outcome of the assessment was also a ‘radar diagram’ which presents areas of strengths and weakness in each dimension of the tool.
- Step 4: The SCIROCCO project facilitated the comparison of the radar diagrams and facilitated the matching of the participating regions for the purpose of the knowledge transfer process. The project then organised the twinning and coaching activities (KT process) between the matched regions. The knowledge transfer could flow between the regions with the same strengths (twinning) as well as between the regions scoring high at particular dimension with the regions scoring low along the same dimension (coaching). The sessions were intended to be organised as study visits, webinars, and through various online tools. In each participating region, two or three local project partners were involved in organising the activities and associated with the KT process for their regions. The project partners also recruited a maximum of five other local experts to participate in the twinning and coaching sessions for their region.

The planned outline for the twinning and coaching sessions, included the following steps. In the first step, the members of each participating region were asked to express their interest for the twinning and coaching activity to be informed by either a) the assessment of the healthcare system (step 2 of the strategy) or b) a selected good practice within the project (step 1 of the strategy). One twinning and one coaching activity was envisaged for each of the five SCIROCCO regions. In the second step, the twinning and coaching process of the receiving and transferring region was intended to be initiated, including introductory webinar(s) between the transferring and receiving region(s). In the third step, a study visit to the transferring region was to be facilitated. The study visits were organised at a location in the transferring region, which was the region/authority acting as the “coaching” partner in the KT process. The receiving region was the region/authority seeking support and know-how in order to deploy a good practice and/or improve a specific aspect of integrated care and acted as the “learning” partner. In the final (fourth) step, a local meeting with the experts in the receiving regions was planned to be organised to reflect on the learnings to be drawn from the twinning exercise. They were also held to agree on the priority actions for the ensuing improvement(s), including policy recommendations and potential impacts. These were then captured in an Action Plan by using a developed template that built on the outcomes of the study visit.

A total of five twinning and coaching sessions, referred to as KT cases (in short cases) were organised including five study visits of which one took place in Puglia, one in Basque Country, two in Scotland, and one in Norrbotten. Three cases included one transferring region and one receiving region, and two cases included one transferring and two receiving regions. In Table 1 an overview and description of the focus on each of the five KT cases is provided.

**Table 1:** Overview of the focus of the five KT cases in the SCIROCCO project

Location of KT cases 1 to 5 (transferring region)	Focus of KT case	Description of the focus of the KT case
1. Puglia	Good practice in telemonitoring	Hospital@Home Good Practice is designed as a technological support for already structured activities in home care. The main objective is to reduce hospitalisation and re-hospitalisation and to improve the quality of care for patients at home. In addition, the objective is to: Reduce the number of patients with heart disease, diabetes and other chronic diseases in the process of instability Activate protected de-hospitalisation Optimise the therapy and diagnosis according to international guidelines. The aim is to implement a new type of telemonitoring, based on continuous collaboration and patient monitoring by different professionals and different users. Patients are telemonitored by their General Practitioners by using the innovative home and health monitoring technological solution (H&H Hospital@Home). This solution is able to detect the main clinical and instrumental parameters in addition to the therapeutic administration, based on oxygen and bronco-aspiration. It is allocated at the patients’ home and it is permanently interconnected with the General Practitioner and/or Specialist, by computer, telephone, tablet and other devices. At the same time, there is a central monitoring room at the hospital for all patients and all devices located at their home. All clinical parameters of patients are stored on a dedicated server, respecting the rules for the respect of privacy. The system allows the healthcare professionals (neurologists, pulmonologist, cardiologists, diabetologists, etc) to monitor and speak with patients remotely. The patients can also activate the visit of the healthcare professionals in their homes. In addition to real-time monitoring of physiological parameters, the healthcare professionals can also monitor the physical and technical characteristics of home device. As a result, it is possible to deliver therapy to the patient remotely.
2. Basque Country	Good practice in advance care planning	Advance care planning (ACP) is a voluntary process of discussion between an individual and care providers about future care, irrespective of discipline. The aim is to guarantee patients’ right to take decisions about their own care as well as to have those decisions respected when time comes. The goal of this Good Practice is to promote ACP approach to the Basque population, particularly to chronic conditions population. The idea is to adjust end of life care to meet patients’ preferences and improve decision making processes. Three stages were defined when designing the Good Practice: Diagnostic stage in order to identify the population that could benefit from the ACP Therapeutic stage in order to develop the intervention Evaluative stage in order to assess both the impact of the Good Practice and the Good Practice itself. The core intervention consists of two individual semi structured interviews with the patient and one or two members of patient family and/or friends. The interviews are carried out by the patient’s General Practitioner (GP) and the community nurse. The first meeting aims mainly at introducing the subject (Advanced Directives) and inviting the patient to reflect on his/her preferences regarding the care. The second interview then focuses on the discussions of the specific issues related to the patient and his/her clinical characteristics and situation. Participants write down an advance directive according to their values, health conditions and preferences. The GP and/or community nurse assist with this process. Every healthcare professional can access the Advance Directive using the Basque Country’s Integrated Electronical Health Record. As ACP is considered an evolutive process, the patient can change opinion and modify its preferences whenever is needed.
3. Scotland	Dimension of the SCIROCCO tool: Innovation Management	Health innovation is an exciting and dynamic area with a range of stakeholders from all sectors working collaboratively to position Scotland as a world leader in health innovation, contribute to a thriving economy and support faster adoption of innovation across health and social care. Innovation is defined in Scotland as the invention, development, production and use of products, medicines, therapeutics, approaches and supporting services which create the opportunity to make major improvements to health and healthcare. Scotland is already recognised as an innovation nation. The recently refreshed 2017 Scottish Life Sciences Strategy sets out strategic priorities for the sector to fulfil Scotland’s ambitions to be a world-leading entrepreneurial and innovative nation. The Scottish Government has outlined its commitment to innovation with recently published Scotland Can Do – Boosting Scotland’s Innovation Performance: An Innovation Action Plan for Scotland (January 2017) and more specifically to health innovation within the Health and Social Care Delivery Plan (December 2016) and recently published Digital Health and Care Strategy (April 2018). A range of initiatives and partnerships are already well established (Innovation Ecosystem) as part of the overall drive to support health innovation and its formalisation in Scotland
4. Scotland	Good practice(s) in Third Sector	The “third sector” in Scotland is made up of non-governmental and non-profit organisations, from grassroot community groups and village hall committees to social enterprises and registered national charities. It is often also described as the voluntary sector, not-for-profit, charity sector, social economy, social enterprise sector, NGOs (non-government organisation) or civil society. The traditional idea of charities as benevolent organisations simply there to help the poor is being replaced by a modern, progressive, third sector which carries out an enormous range of activities to improve people’s lives. It does it by: -Supporting people through social care, health services and employability programmes; -Empowering people by campaigning and advocating for minority and disadvantaged groups in our society; -Bringing people together through social activities, local clubs and community centres; -Enabling better health and wellbeing through medical research, addiction services, sport facilities and self-help groups; -Improving our environment through conversation of our land and heritage, and regeneration of our communities. In Scotland, there is a legal framework in place for the engagement of third sector in the provision of integrated care. The Public Bodies (Joint Working) (Scotland) Act 2014 is the legislative framework for the integration of health and social care services which requires the integration of the governance, planning and resourcing of adult social care services, adult primary care and community health services and some hospital services. Other areas such as children’s health and social care services, and criminal justice social work can also be integrated.
5. Norrbotten	Dimension of SCIROCCO tool: Information and eHealth	A need to introduction technology enabled solutions is widely recognised among all stakeholders involved. There is a clear plan and strategy in place to support widespread implementation of eHealth services. Patients are widely supported and encouraged to manage their own care and participate actively in the decision-making process through the access to electronic health records and relevant health information. National ICT solutions to increase patients´ access to their medical records have been developed and implemented in Norrbotten Region on a large scale. There are also national ICT solutions to support patients´ participation in the management of their own care but not fully implemented yet. A share of patient related information between different care providers is facilitated at the regional level. Norrbotten Region has also very well progressed with building the ICT solutions on existing platforms and infrastructure and has thus created new services to empower patients and ensure their ability to participate in the decision-making on their care as well as supporting self-care. However, scalability of these solutions still remains the issue.

### Data collection

Several qualitative research methods were used to collect data from each of the five cases, involving five focus groups and several documents; including seven action plans, five study visit programmes and one project document.

#### Focus groups

Between June 2018 and September 2018, a total of five focus groups were conducted after the study visits took place. The purpose of the focus groups was to collect the (shared) experiences of the participants and details on the SCIROCCO KT activities in each of the five cases. By undertaking focus groups, several perspectives of the participants could be collected while encouraging the participants to question each other [17] and to explore each other’s views, which can lead to a detailed exploration of ideas [18]. They were held in suitable venues in the specific locations where the study visits were organised All the experts who participated in the study visits were invited to participate in the focus groups. These experts were recruited by the local partners based on their experience with the good practice or expertise that was the subject of the study visit. Some experts of the transferring regions were involved in the study visit by providing presentations and were not involved in the complete study visit, those experts were not participating in the focus groups. Each participant received a detailed programme of the study visit, which included details of the planned focus group at the end of the visit. The characteristics of the focus groups are presented in Table 2.

**Table 2:** Characteristics of the focus groups

Location of study visit and focus group (transferring region) (cases 1 to 5)	Subject of focus group	Local stakeholders involved (n)	Gender	Job role	Total number of participants	Facilitator(s)	Date of interview
1. Puglia	Experience in study visit on GP in telemonitoring	Olomouc stakeholders (receiving region) (5)	Male: 3 Female: 2	Medical Expert: 2 Telemedicine expert: 2 Project manager: 1	14	WP3	14th of June 2018
		Scotland stakeholders (receiving region) (6)	Male: 1 Female: 5	Manager: 3 Government advisor: 2 Director: 1			
		Puglia stakeholders (3)	Male: 2 Female: 1	Consultant: 1 Regional manager: 1Director: 1			
2. Basque Country	Experience in study visit focused on GP in advance care planning	Norrbotten stakeholders (receiving region) (3)	Male: 0 Female: 3	Project director: 2 Improvement officer: 1	6	WP8	12th of June 2018
		Basque Country stakeholders (3)	Male: 2 Female: 1	Director: 1 Project manager: 1 Nurse: 1			
3. Scotland	Experience in study visit focused on innovation management dimension of the SCIROCCO tool	Norrbotten stakeholders (receiving region) (5)	Male: 1 Female: 4	Communication officer: 1 Innovation Developer: 1 Project director: 2 Improvement officer: 1	8	WP3, WP8	26th of June 2018
		Scotland stakeholders (3)	Male: 0 Female: 3	Director: 2 Manager: 1			
4. Scotland	Experience in study visit focused on GP in Third Sector	Basque Country stakeholders (6) (receiving region)	Male: 2 Female: 4	Director: 1 Project manager: 2 Government advisor: 1 Program manager: 2	13	WP3	5-6th of Sept 2018
		Puglia stakeholders (4) (receiving region)	Male: 2 Female: 2	Regional manager: 1 Consultant: 1 Project coordinator: 1 Director: 1			
		Scotland stakeholders (3)	Male:0 Female: 3	Project coordinator: 1 Manager: 2			
5. Norrbotten	Experience in study visit Norrbotten focused on information and eHealth dimension of the SCIROCCO tool	Olomouc stakeholders (receiving region) (4)	Male: 3 Female: 1	Telemedicine expert: 1 Project manager: 1 Director: 2	8	WP8	12-13th of Sept 2018
		Norrbotten stakeholders (receiving region) (4)	Male: 3 Female: 1	Project director: 2 Improvement officer: 1 Nurse: 1			

The questionnaire for the semi-structured focus groups was developed in collaboration with members of two other work packages (WP5 and WP8) who were active partners in the SCIROCCO project. WP8 was focused on collecting the lessons learned on the process of KT using the SCIROCCO tool and WP5 was oriented towards the design of the SCIROCCO tool. The framework for KE of Ward et al. was used to guide the topic list development to collect the experiences on the SCIROCCO KT activities for this study [10]. The full questionnaire is available via the corresponding author.

The focus groups were alternately facilitated by one member of WP3 and two members of WP8. The three moderators possessed a minimum of a master’s degree and experience in qualitative research. At the start of the focus groups, the moderators provided an introduction to the focus group and themselves, explaining the purpose of the focus group, and requesting the participants to sign the informed consent form (see ethics statement). All participants received an overview of the focus group questions at the beginning of each study visit. Each focus group lasted approximately 1 hour and was audio-recorded and transcribed verbatim.

#### Documents

To examine the details on how the SCIROCCO KT process unfolded within the project, document data including the action plans, study visit programmes, and a project document on the twinning and coaching methodology were also collected between April 2018 and January 2019. The action plans contained descriptions on the outcomes of the study visits. The plans were co-designed by the transferring and receiving regions. The study visit programmes included the experts involved in the study visit, and the programme for the 1 or 1.5 day study visit. A total of seven action plans, five study visit programmes, and one project document were collected.

#### Data analysis

The analysis of the data was guided by the two frameworks of Ward (et al.) [7, 10]. All data (the focus groups transcripts and collected documents) were analysed using a directed and conventional qualitative content analysis approach [19, 20]. The aim of combing the two methods was triangulation for the convergence and confirmation of findings [21]. A coding scheme, including the five KE components and themes derived from the framework and the four knowledge mobilisation elements and categorisations, was used during the coding process. In Table 3, a short overview of the definitions used in the analysis of the components including the themes and questions of mobilisation is presented. By using the directed approach, all data were reviewed for content and deductively coded per KT case according to the categories of the coding scheme [22], to achieve fewer content-related categories [23]. To ensure a homogeneous interpretation of the data, a content check was performed. Two researchers (LG, HV) checked one focus group independently and compared them. The results from this coding process were discussed among the researchers and any disagreement was resolved until consensus was reached.

**Table 3:** Descriptions of the five components of KE by Ward et al. [10] including the themes and elements of knowledge mobilisation [7]

Problem definition involved: identifying, clarifying, focusing, reviewing and evolving the problem over time.	
Context involved: exploring, discovering and revealing context which consists of the personal, interpersonal, organisational, and institutional characteristics relevant to transferring knowledge into action.	
Knowledge involved: locating the knowledge, classifying the knowledge, assessing the knowledge, tailoring the knowledge, usability of the knowledge/practical limitations, and whose and what knowledge?	
Intervention involved: negotiating KT roles and responsibilities, clarifying the type of intervention to be used (information management, linkage, decision/ implementation support, capacity development), integrating the intervention, making the intervention iterative, and why and how mobilise knowledge?	
Use involved: deciding how the knowledge will be used (knowledge was used in a range of different ways: directly i.e. with little modification), conceptually (i.e. to change opinions) or politically (i.e. to confirm or challenge practices or policies), considering the practicalities of use, spreading knowledge to others and sustaining knowledge use.	

Although the analysis was primarily deductive, when the data did not fit the concepts of the coding list, the codes on the scheme were adjusted accordingly. Some codes on the coding scheme did not directly fit the data and we used the conventional approach to be able to adjust some of the codes to fit the data. With regards to the “knowledge” component, besides looking into the type of knowledge offered by the transferring regions, the type of knowledge needed by the receiving regions was also included as a code. In addition, for the intervention component, “to be used” was included after the type of intervention, as the actions were indicated as proposed actions.

Since this study took place within a project facilitating KT between known transferring and receiving regions, we chose to add an additional category under “Whose knowledge is being mobilised?” by including “knowledge receivers”, referring to the knowledge recipients involved as the experts of the receiving regions. An elaboration to the description of one group of “knowledge donor/receiver” was added and included policy makers to the category “Decision makers” to better match it to the interpretation of this study. Furthermore, the categories of “Why knowledge is being mobilised” were slightly adjusted.

The coding process took place using QSR International’s NVivo 12 software. After all the data were coded, the final data analysis phase was a cooperative effort between LG and HJMV. The analysis was an iterative process: it was made up of initial analyses by LG, followed by discussions between LG and HJMV, and further analyses and discussions in order to identify concordant and discordant themes. Any disagreement was resolved through discussion.

The validity of the findings was ensured by the examination of several cases and the use of triangulation as data from focus groups and documents were collected to inform the concepts of the knowledge transfer process [24]. This established a more thorough and multi-faceted examination of the KT processes in SCIROCCO than could be gained from any single method or single case.

## Results

The structure of the findings follows the five components of KE, including the distinct themes describing the nature of KE. The four questions derived from the knowledge mobilisation framework clarify some of the components. The two questions on whose and what type of knowledge is being mobilised are included under knowledge. The why and how questions on knowledge mobilisation are included in the intervention component.

### Problem

At several points in time during the KT process, attention was paid to the challenge that the receiving regions chose to address. Two different approaches were designed for carrying out the KT activity.

The first approach (a) was informed by the maturity assessment of the healthcare system using the SCIROCCO tool. The outcomes of the assessment provided insight into the strengths of, and challenges for, integrated care in the region. Hereafter the region chose one domain for improvement. It sought support from another region which had previously demonstrated a significant progress in the corresponding domain (as shown in the outcomes of the maturity assessment). Within the project, two cases (3 and 4) focused on improving a specific domain/aspect of integrated care using the first approach.

In the second approach (b), the problem identification focused around a strategic interest of a region in one of the good practices which were selected by other regions in the project. After the region expressed their interest, the requirements of that good practice, for it to be adopted and transferred, were assessed using the SCIROCCO tool. Then, the receiving region assessed the maturity of the healthcare system for the adoption of the good practice using the SCIROCCO tool. After the regions explored the requirements of the healthcare system to adopt the good practice, the twinning and coaching process was initiated. Within the project, a total of three cases (1, 2 and 5) focused on the second approach.

After the regions were matched, they used different approaches to clarify the problem/challenge of the adopting regions before the study visit. One transferring region explicitly indicated to be in contact with the adopting region prior to the visit (case 5). In the other cases, the regions involved did not provide details on how the preparations on clarifying the challenge of the receiving regions was conducted. Participants in case 2 mentioned that the study visit would have benefitted from more preparation.

Clarifying/focussing on the challenges of the regions occurred during the study visits. In the programme of four out of five study visits, explicit time was scheduled to discuss the rationale for the twinning and coaching between the transferring and receiving region(s). The challenges of the adopting and transferring regions were sometimes also mentioned during the focus groups. Some respondents talked about reviewing the challenge experienced by their region, based on the knowledge they had received during the study visit. Quotes are presented in Additional file 1.

In the final step of the KT process, the challenge of the regions was described in the action plans by the regional project leaders of SCIROCCO (details are provided in Additional file 1). For all five cases, the background of the problem was identified as being part of a broader process for change and/or improvement of the health and social care systems. Almost all representatives of regions, acknowledged that the sustainability of their health and social care systems is becoming a challenge. Hereafter, the problem was more focused towards the subject of the KT activity: a short description of these challenges is presented in Additional file 1.

No direct observations were made where the problems in the regions evolved over time. In two cases, however, respondents mentioned that the tool could be used to track progress over time (in case 1 and 3).

### Context

Exploring, discovering and revealing contextual characteristics was a central part of the five KT activities within the SCIROCCO project. This activity was supported by using the SCIROCCO tool. In the two study visits (in case 3 and 4), using the first approach, a facilitated discussion was organised that compared the self-assessments of the transferring and receiving region. For each dimension, this included the identified features of the health care system. The features were concrete attributes of the environment that are needed for improvement. The receiving regions explored what they needed to change in the local environment in order to enable the improvement of that domain in their local context. They also considered whether improvement in this specific aspect on integrated care related to other dimensions of the SCIROCCO tool. These aspects were later captured in the relevant action plan.

In the second approach, the KT was informed by the assessment of maturity requirements, first, of a good practice for adoption and, second, of the health care system of the receiving region. A maturity requirement is a feature that a good practice needs the environment for it to be implemented. In two out of the three study visits, a discussion was facilitated focusing on what would be the requirements of local health care systems to transfer the good practice (one did not take place because of lack of time). The outcomes of these discussions informed the development of the action plan.

In Additional file 2, the classification of contextual dimensions made by the receiving regions is presented based on the assessment of the extent to which the transfer of knowledge per contextual dimensions was regarded feasible to the local context. The organisational or professional contextual structural characteristics, were sometimes indicated by regions, and are also presented in Additional file 2.

### Knowledge

#### SCIROCCO’s KT procedure

The SCIROCCO tool and project activities supported regions in locating the knowledge. The tool and project activities assisted in the matching of regions and the further KT processes. Assessing the relevance and usefulness of knowledge by the experts of the receiving regions occurred during facilitated discussions in six out of the seven study visits. Based on the contextual dimensions and features, the experts assessed whether transferring the learning about the good practice or the learning about a dimension was feasible in their region’s context. This was done by indicating whether transferability was feasible (yes or no). When it was considered feasible, this was further assessed by indicating whether this required little or much effort or adaptations.

After looking into the feasibility, a further selection of the knowledge was made. In the action plans, the receiving regions listed a maximum of three prioritised features to be considered for the transferability of learning about a good practice, or an improvement in dimension in the receiving regions’ local context. Hereafter, the adopting regions described per listed feature the suggested adaptations to their local context to enable the creation of conditions for the adoption of the learning from the good practice or dimension (Additional file 2). The suggested adaptations can be understood as tailoring knowledge.

#### Type of knowledge

##### Transferring regions

The knowledge shared by the transferring regions came from different sources, including presentations and discussions among the experts from the transferring and receiving regions. In four cases, knowledge also came from practical site visits. Furthermore, the transferring regions provided information in the action plan of the receiving regions. The knowledge shared among the regions came from a mix of “knowledge donors”, who were involved in the KT activities and differed case (Additional file 2). Only one transferring region, case 3, included members of the public who were acting as, or on behalf of, their communities and people in receipt of services (i.e., service users). Furthermore, the type of knowledge which was offered by the transferring regions during the five organised KT activities within the SCIROCCO project varied per case (Additional file 2). In three study visits, scientific/ factual knowledge was shared in the form of data on the performance of the practice shown during presentations or was described in the action plans. Technical knowledge was shared during the presentations in the study visits. The sharing of technical knowledge and practical wisdom were reflected in sharing experiences with the experts of the transferring regions during both the discussions and demonstrations in the site visits.

##### Receiving regions

The participants from the receiving regions, who were regarded as the “knowledge receivers”, included a mix of experts, composed of project members of SCIROCCO and invited regional experts (Additional file 2). In all cases programme and programme developers were involved as experts. Only one receiving region included a member of the public acting as, or on behalf of, the person’s community and people in receipt of services.

With regards to the type of knowledge, the adopting regions described in their action plans per listed feature the suggested adaptations/changes of the features to their local context. The type of knowledge which was of interest for the regions is shown in Additional file 2. The type of knowledge needed categorised as scientific/factual knowledge, were described by two of the receiving regions and included the feature of the SCIROCCO tool entitled Evaluation Methods.

The need for technical knowledge was noted in all the seven receiving regions. Technical knowledge was about developing (implementation) plans/mechanisms (enabling adoption), reforming/developing legislation and embedding learning though education and training and included different “dimensions/features.” The last type of knowledge needed, practical wisdom, was found in five regions. The need for practical wisdom included raising awareness about a new way of working, increasing public awareness, and demonstrating benefits of the good practice or improvement in an aspect of integrated care. Features that emerged included Removal of Inhibitors, Citizen Empowerment, Readiness to Change, Innovation Management and Information and eHealth services (these five are among the 12 dimensions that populate the SCIROCCO tool).

### Intervention

For the intervention concept, a distinction is made between the intervention consisting of the SCIROCCO project itself and the priority actions of the adopting regions as described in the action plans. These two sorts of interventions are described separately below.

#### SCIROCCO intervention

When focussing on the SCIROCCO project, three types of KT interventions were reflected in the methodology for twinning and coaching sessions. Starting with information management, the SCIROCCO project supported the regions in finding the knowledge in another participating region. Linkage and exchange occurred as the five KT activities organised by SCIROCCO included study visits to bring together the matched regions. All study visits included presentations from the transferring region. Almost all accommodated discussions among the regions based on comparisons of the self-assessments and some involved practical site visits. Finally, capacity building was facilitated by helping the regions to reflect on the possibility to transfer and adopt the learning about the good practice or dimensions to local settings, by drawing up an action plan following the study visit. On the negotiating KT roles and responsibilities within the SCIROCCO project, the SCIROCCO local project members were part of the KT activities representing their regions and they invited several types of regional experts to be part of the KT: details on these types of experts are presented in the “knowledge” section. The knowledge mobilisation technique used by SCIROCCO can be categorised as “making connections between knowledge stakeholders and actors by establishing and brokering relationships.”

The participants provided feedback on the SCIROCCO study visits: a short overview of their feedback on these visits is presented here.

The use of the SCIROCCO tool as part of the knowledge transfer activity was considered according to respondents in case 1, 4 and 5 as supportive in focussing/structuring the discussion during the study visit between the regions. Two experts (in cases 1 and 2) suggested to edit or the need to add elements in addition to the tool. Some experts indicated experiencing issues with the language of the tool (in cases 2,4,5). Overall, participants observations covered the usefulness of study visits especially when they including a practical (real life) trip and/or presentations (in cases 1, 2,3); an appreciation of the collaboration and process involved (in cases 1 and 3) and of the role that the site visits played in mutual learning (in case 4). Organisationally, the length of time sometimes spent on a study visit or on its preparation could, in some cases, have been lengthened (in cases 1, 3,5).

#### Interventions to be used by receiving regions

During the study visits, there was time for the regions to discuss and clarify possible interventions to transfer the learning/knowledge to their local contexts. This means that the adopting regions discussed what changes/improvements were needed to enable the transfer of the good practice or the improvement in an aspect of integrated care in their local environments. (This is also reflected in the knowledge needed by the regions as presented under “knowledge” section). Once back home, these processes were further clarified and written down in the regional action plans in the form of priority actions. An iterative process of selecting an intervention by the regions could not be observed. At the end of their action plans, the regions listed the actions proposed to enable conditions for adopting the learning of the good practice or to enable conditions for improvement of innovation management in the local context. The actions included objectives, anticipated outcomes, and policy implications. The priority actions of the regions are categorised under the type of intervention to be used and are presented in Additional file 3.

The type of intervention categorised as capacity development was described by all the regions. It involved raising awareness among professionals or citizens when certain improvements are needed. It concerned e.g. engaging professionals or embedding/improving education and training. Strengthening/improving or positioning several roles as part of the intended change were also considered part of capacity development. Linkage, as intervention, emerged as engaging/involving several stakeholders or joining efforts among actors, and encouraging participation and partnership building in the intended change. Decision and implementation support were reflected when receiving regions referred to developing plans or strategies for implementation, extending or scaling-up initiatives, or embedding elements in regulations or policies. Information management came up in a few regions, indicating the collection of data/information on the change and publishing data.

The study team also looked whether the actions could be categorised under the “How mobilise knowledge?” concept. However, since the action plans refer to “proposed” actions and policy “implications”, the actual implementation of these plans was out of the scope of the project. As a result, it was not possible to categorise the “How mobilise knowledge?” concept for these actions.

Attention was paid to negotiating KT roles and responsibilities in the action plan, as the receiving regions were encouraged to think of who would be the (future) responsible actors for the priority actions. Six of the seven regions pointed out the responsible actors (see Additional file 3). Furthermore, the regions indicated policy implications for the intended actions, which can be considered as a form of integrating the intervention/priority action in their local context.

### Use

A range of ways of how the knowledge will be used could be retrieved from the action plans (see Additional file 3). The knowledge transferred during the twinning and coaching sessions is expected to be used mainly conceptually (i.e. to change opinions) and politically (i.e., to confirm or challenge policies) by the receiving regions. The receiving regions indicated policy implications for the proposed priority actions. Some regions indicated that they have a range of policies in place supporting the actions, while other regions were in the middle of developing them or opted for expressing the need for policies or strategies to support the action. The policy implications indicated are presented in Additional file 3 under “knowledge used politically.” These policy implications, including the request to think of the responsible actor(s) and anticipated duration of the action, can be considered as SCIROCCO’s way to support the receiving regions to think of sustaining and spreading knowledge.

The receiving regions also indicated the practicalities of knowledge use, as sometimes regions indicated during the assessment of knowledge, that the knowledge would not be feasible to transfer (see “context” section). Practicalities are also considered in the action plans, where the adopting regions described the benefits and opportunities of the adoption of the good practice or of improving a particular dimension in their region. These are summarised in Additional file 3.

The categories on “why knowledge is being mobilised”, reflected in these practicalities, are also presented in Additional file 3. The reasons for mobilising knowledge between the regions are found mainly to be a mix of “To (further) develop new policies, programmes and/or recommendations”, and “to change practices and behaviours.” Also, a few regions were planning to use the knowledge “To adopt/implement transferring regions ideas on practices and policies.

## Discussion

This multi-method study had the purpose to provide insights into how the processes of KT, facilitated between five European regions unfolded as part of the SCIROCCO project, aimed at the transfer and scaling-up of successful integrated care initiatives. To explore this aim, data were collected within the project by conducting focus groups and examining the content of project documents.

The two frameworks used to guide this study were found to be useful for analysing the KT processes. Moreover, the SCIROCCO project appeared to have designed an extensive approach for the KT process among five participating regions. It was found that the five components (including the themes) of KE [10] could to a large extent be identified in the developed methodology for the SCIROCCO twinning and coaching activities. Furthermore, the four questions and accompanying categories of the framework for knowledge mobilisers [7], were also identified to a large degree and provided additional insights in the SCIROCCO KT processes.

These key findings are discussed below in terms of the problem, context, knowledge, intervention and use before the strengths and limitations of the study are explored.

### Problem

In all five cases, on several occasions in the KT process, attention was paid to problem definition. Evolving the problem definition over time could not be observed in the five cases of this study. There are two possible explanations for the periodicity of this treatment of problem definition. The first explanation may be the difference in both studies in the type and intensity of interactions between the facilitating party (called the “knowledge broker” by Ward and colleagues [10]) and the receiving “team.” In the study of Ward et al. the knowledge brokering activities “were driven by the teams’ own problem-solving processes [10].” In contrast, the outlined step-based design of KT processes within SCIROCCO were implemented within the scope of a project, offering limited options by a time-bound and defined programme. Therefore, it might not have been possible to allow sufficient time and space for the problem to evolve. Indeed, some regions indicated the potential for the SCIROCCO tool to be used in the future to track changes over time. As Ward et al. observed “that an inability to revise and evolve KE problems can hamper the desired change process [10]”, it is advised to allow for the evolution of problem in the design of the SCIROCCO-based KT processes in the future.

The second possible explanation is that, in the study of Ward et al., the knowledge broker participated in the KE process of three teams over a period of 10–15 months and collected observational field notes [10]. In our study of the experiences in SCIROCCO, data were collected on four occasions and no direct observations were made during the transfer of knowledge. This could mean that we were unable to detect the evolution of the problem over time. Furthermore, this could also explain the fact that insufficient data were gathered to enable a comprehensive insight in step 2 of the KT approach, when the regions were matched and prepared themselves for the knowledge transfer before the study visits took place.

### Context

Contextual characteristics were specifically considered in the five KE cases by using the SCIROCCO tool to inform three steps in the KT processes. Not all themes identified by Ward et al. as being related to context, were reflected in the SCIROCCO approach [10]. The contextual characteristics within the SCIROCCO project are more focused on the macro health care system related to integrated care; however, some organisational and professional structural characteristics were reflected in the suggested adaptations to context addressed by some receiving regions. Ward et al. indicated that their findings suggest “that KE is a social and political rather than behavioural phenomenon which involves professional identities and norms in addition to individual beliefs” and that “fractions within a group may instigate KE as part of a strategy of contesting professional norms and identities [10].” This phenomenon is observed in SCIROCCO by the fact that several receiving regions indicated the requirement to raise the awareness of professionals for the need for change or to the benefits of change. Moreover, Ward et al. suggest that “knowledge translation approaches need to focus beyond individual behaviour or specific organisational characteristics [10].” Although they are focused on integrated care, the wide scope of the contextual dimensions which are part of the KT process in the SCIROCCO approach could be interesting to consider either in the framework of Ward et al. or in other KT processes [10]. Vice versa, the focus on personal, individual and interpersonal characteristics could be useful, when using an approach like SCIROCCO, when receiving regions would be interested in transferring the knowledge retrieved to the level of actual practice.

### Knowledge

All five cases were actively supported by the SCIROCCO project in locating, assessing and tailoring knowledge during several steps of the knowledge transfer process. Ward et al. observed in their study that locating and tailoring knowledge was “rarely instigated by the knowledge broker” and that the teams “classified and selected knowledge in relation to their professional backgrounds and training and that these preferences are amenable to change through reflexive action by team members [10].” In contrast, these processes were outlined in the SCIROCCO approach. The professional background of experts could have played a role in the classification and selection of knowledge within SCIROCCO and, in the study visits, the facilitated discussions may have supported reflective action among the experts. However, since data for this study were only collected through focus groups, where reflection is an actual part of the data collection technique, and no observations were made during the discussions that took place in the study visits, the influence of the various professions on changing preferences and reflexive actions could not be observed. Nonetheless, in the SCIROCCO KT process, there was indeed time and space for discussions among different professionals, which suggests that support was offered for Ward et al. advised “naturalistic processes of reflexivity and discrimination [10].”

The knowledge offered by the transferring regions came from a mix of knowledge donors, which were identified according to the categories of Ward [7], and several types of knowledge were offered. In contrast to Ward, we did focus on knowledge receivers since this would provide insight into the type of experts involved in the knowledge change process within SCIROCCO [7]. Ward indicated that focusing on knowledge receivers “suggests that knowledge is a product which is to be translated into practice, […] and is at odds with observations of the fluid, multidirectional nature of knowledge mobilisation [7].” In the process of knowledge transfer between several international regions, designated experts to participate in the process are needed. This selection does not mean that the KT process of SCIROCCO did not consider the potential for a fluid, multidirectional nature to the KT process, rather, the diverse types of experts were encouraged to think of how to deploy knowledge in the regions and to name responsible actors.

### Intervention

The themes identified by Ward et al. in the “intervention” component were found to be facilitated within the SCIROCCO project [10]. Regions having discussions on “making the intervention iterative were not observed.” Possible reasons, reflected by the scope of the SCIROCCO project or the lack of direct observations made, are elaborated on above under “Problem.”

Ward et al. found in their study that “many of the KE activities which we observed were an integral part of the process of change in which the teams were engaged” and argued that “the development of […] knowledge translation interventions could begin by focusing on these naturalistic KE activities [10].” They suggests that this could increase the willingness of members to engage with KE interventions and would also make them more easily conceivable in the absence of resources for external assistance.

All regions involved in the KT activities of SCIROCCO were found to be engaged in a broader process of change. However, the KT process of SCIROCCO can be regarded as an “add-on” intervention (using external resources and skills) to facilitate the KT process between regions. In addition, the SCIROCCO project also focused on the development of the tool to facilitate transfer among regions, which required additional resources. Nonetheless, the SCIROCCO approach seems to correspond to elements of the natural processes of KE and to be open to a wide range of sources and different types of knowledge resulting in a variety of types of interventions intended to be used. The general trend among the participants in SCIROCCO was of a positive experience derived from the study visits. This suggests that the participants were willing to engage in SCIROCCO’s KT process. The approach of matching regions will, however, require external resources. Furthermore, the tool seems to be a useful tool in providing support to the regions to identify the problem, locate, clarify and assess knowledge and possible interventions during the KT processes. This indicates that the SCIROCCO tool has shown potential in the knowledge transfer process.

### Use

Conceptualisations of knowledge use were found to be part of the SCIROCCO KT activities and included various forms of intended knowledge use in the five cases. Ward et al. suggest in their study that “KE can be understood as a dynamic and fluid process which incorporates distinct forms of knowledge from multiple sources [10].” The incorporation of distinct forms of knowledge from multiple sources are reflected in the SCIROCCO approach and the dynamic, fluid process could to a certain extent be observed. Some or sometimes all the components have been shown to occur simultaneously at different steps within the KT processes of SCIROCCO. However, a fluid process was not always reflected in the processes explored in this study. There are at least two potential explanations for this. This could be due to the limitations in data collection (as described under Problem). Another explanation lies in the outlined programme of SCIROCCO’s KT process, this could have resulted in a more “linear” approach which could have compromised the fluid processes.

Knowledge mobilisation models have been found to focus on how change occurs, to lack practical utility and not to focus on the content of change activities [14, 25]. The findings of this study contribute to providing concrete evidence to counterbalance the previous lack of practical insight into the specific methods of KT initiatives. Altogether, we consider the insights gained into SCIROCCO’s unique methodology for KT and how it unfolded in practice valuable.

Furthermore, it is questionable whether there are any insights available from other studies about looking into practical knowledge transfer between international regions, as many studies focus on knowledge transfer between research and practice [26–28] . The insights obtained in this study are, specifically, compelling for other regions that are interested in SCIROCCO’s KE process or, more generally, in the exchange of knowledge in the field of integrated care.

### Strengths and limitations

There are three main strengths to this multi-method study. First, this study was guided by two frameworks which supported the data collection and analyses. This use of frameworks is important since the transfer of knowledge and the scalability of elements of integrated care initiatives to other organisations/regions lacks clarity and poses great challenges, and the literature supported us in obtaining a better understanding. Second, the focus groups enabled a depth of coverage of knowledge transfer issues, and by conducting document analysis, breadth was also achieved. The multi-method qualitative nature of this research enabled some practical insights into a KT transfer initiative and demonstrates what the approach yields for participants. Third, the collected data of the exchange of knowledge between several diverse European regions, in an international context, enabled obtain insights to be obtained which are likely to be applicable to other contexts.

There are three main limitations to the study. First is the fact that data were collected on four occasions, and no direct observations were made during the exchange of knowledge within SCIROCCO. The methodology for the KT process was developed and planned on short notice within the framework of a wider project, which had dealt with delays and deadlines. Although we were partner members the SCIROCCO project, which enabled us to follow the project closely, we had to consider the work timetabling of other project activities and thereby make instrumental choices about the data collection. This constrained the ability to collect data to cover all the potential components reflected in the KT process. The second limitation is that since the researchers were partner members of the SCIROCCO project, there is a risk of bias in the focus groups. Through the course of the project, the researchers continuously reflected on the methodological decisions and their own role in the research process and ensured their independent role as researcher by not interfering with any project activities except the evaluation activities. A third limitation which needs to be addressed is the practical use of the frameworks of Ward (et al.) [7, 10].. Ward et al. stated that the framework of KE has “elements [that] need further examination” but suggested that it can “act as a starting point for exploration and evaluation [10].” For the second framework, Ward mentioned that “although the framework does not offer an easy set of methods or tools for evaluating knowledge mobilisation initiatives, it can provide some basic building blocks for determining and planning suitable evaluation strategies [7].” Despite the limitations of the practical applicability of the frameworks, given that there is a lack of available tools and mechanisms for evaluating knowledge mobilisation projects, to our understanding, these were the most comprehensive KT frameworks which were available, which fit the study. Some elements of the Ward et al. [10] and Ward [7] frameworks did not fit the specific knowledge change process of SCIROCCO, as the focus of the studies differed. Furthermore, some descriptions of concept were broad. Therefore, we did adjust some concepts and provided our own interpretation to some concepts.

## Conclusion

This multi-method study provides new insights into how the KT processes unfolded as part of a European financed project aimed at the transfer and scaling-up of successful integrated care initiatives.

When compared with two frameworks which focus on KE and knowledge mobilisation, the SCIROCCO project seems to have used an extensive approach to the KT processes implemented in several European regions. The insights obtained could support other regions interested to use the SCIROCCO tool and processes in terms of what to consider during KT with another region, especially in order to improve the local conditions that may enable the adoption and scaling-up of integrated care. Due to its limited duration, the SCIROCCO project did not address the implementation and monitoring of the set of regional action plans, which were written by the five regions involved to capture the learning derived from twinning and coaching sessions. The implementation of the action plans would benefit from an iterative implementation process which could also be useful for interested regions. Furthermore, additional evaluation research is recommended to gain insight into the implementation processes of the plans and the monitoring of their implementation progress in the regions.

## Supplementary information

**Additional file 1.** Overview of content problem per knowledge transfer (KT) case.

**Additional file 2.** Classification of contextual dimensions (dimensions are described in the SCIROCCO tool) and organisational or professional contextual characteristics, type of knowledge mobilisers and receivers and type of knowledge transferred or needed during the knowledge transfer activities of SCIROCCO.

**Additional file 3.** Type of Intervention, including responsible actors and Use of knowledge as indicated by the receiving regions.

## Data Availability

The datasets used and analysed during the current study are available from the corresponding author on reasonable request.

## Abbreviations

KE: Knowledge exchange
KT: Knowledge transfer
SCIROCCO: Scaling Integrated Care in Context

## Footnotes

1 The SCIROCCO tool consists of 12 dimensions which represent the range of activities that need to be managed in order to deliver integrated care. These 12 dimensions include: Readiness to Change, Structure & Governance, Information & eHealth, Standardisation & Simplification, Finance & Funding, Removal of Inhibitors, Population Approach, Citizen Empowerment, Evaluation Methods, Breadth of Ambition, Innovation Management and Capacity Building. The maturity of health care system for integrated care or the maturity requirements of good practices in integrated care are assessed by considering each dimension and allocating a measure of progress or ‘maturity’ (on a 0–5 scale) to each dimension. The scales include the maturity indicators and reflect the basic indications to look for when assessing the current situation of the maturity of health care system for integrated care or the maturity requirements of good practices. After the assessment, a simple graphical representation (i.e. spider diagram) of status can be derived which reveals areas of strength, further attention and improvement in each of the 12 dimensions.
