# Supplementary material for: The transfer of knowledge on integrated care among five European regions: a qualitative multi-method study
Source: BMC Health Serv Res. 2020 Jan 3;20:11. doi: 10.1186/s12913-019-4865-8 (PMC6942405; doi:10.1186/s12913-019-4865-8)
Supplement: Supplementary file 1 — Additional file 1. Overview of content problem per knowledge transfer (KT) case. [file 12913_2019_4865_MOESM1_ESM.docx]

Additional file 1 Overview of content problem per knowledge transfer (KT) case

| Concept Problem | Case 1 Puglia | Case 2 Basque Country | Case 3 Scotland | Case 4 Scotland | Case 5 Norrbotten |
| --- | --- | --- | --- | --- | --- |
| Focus of KT case | Good practice in telemonitoring | Good practice in advance care planning | Dimension of the SCIROCCO tool: Innovation Management | Good practice(s) in Third Sector | Dimension of SCIROCCO tool: Information and eHealth |
| Transferring region | Puglia described the next five challenges that Hospital@Home good practice addresses in their region:  ‘’Reduction of the number of patients with chronic diseases in the process of instability  -Reduction of hospitalisation and re-hospitalisation  -Activation of protected resignation  -Optimisation of the therapy and diagnosis according to international guidelines  -Promotion of the integrated management of hospital and community.’’ | The Basque country described the next challenges that advanced care planning (ACP) good practice addresses in their region are:  ‘’-Improvement of the quality of end-of-life care, respecting patients’ preferences  -Promotion of citizen participation in shared decision-making  -Improvement of care communication between patients and careers  -Increase of health, social workers and caregivers’ competences regarding ACP  -Increase of patient’s competences to make end-of-life/care-related decisions’’  One respondent from the Basque Country indicated the following:  R1: “[...] this kind of sharing and collaboration, it helps you to reflect on many things you are doing, and what other regions are doing with the same issue. And it helps a lot to keep on going and keep on sharing.’’  R1: “In my case what we need if we go to the region, probably we are quite good at structuring things, saying what regulations are what the managers consider. I am not sure that we are good at what is more soft.” | Scotland described that ’’a key driver towards integration and the engagement of third sector in the provision of integrated care has been the projected increase in demand for health and social care as a result of an increasingly ageing population, in particularly those who will be aged 75 and older. […] Integration across the health, social, housing and third sector is seen as a way to make more efficient and effective use of limited resources and is believed to be central to the challenge of improving outcomes for patients and service users.’’ | Scotland described that ’’innovation is the key to delivering a new model of healthcare that meets the challenges and expectations of the modern society. […] When properly targeted and applied, innovative ideas and technologies can transform patient care within the National Health Service (NHS) and other stakeholders involved. As such, innovation helps to deliver patient care while simultaneously improving quality and efficiency, releasing savings through increased productivity.’’ | Norrbotten described that the ’’introduction of information and communications technolog y (ICT) solutions, including eHealth services is the key to delivering a new model of healthcare that meets the challenges and expectations of the modern society. […] When properly designed and applied as routine care, ICT solutions and eHealth services can transform patient care in the Region. As such, implementation of eHealth solutions helps to deliver patient care while simultaneously improving quality and efficiency, releasing savings and demands through increased productivity.’’ |
| Receiving region 1 | Scotland described in their action plan the challenge of “the mainstreaming of technology enabled care initiatives into the routine care. And that Scotland is very keen to explore and learn […] in the area of adoption of technological solutions in the routine care.”  Two of the respondents from Scotland indicated the following:  S1: “In Scotland we have services that use telemonitoring, but we don’t have well developed services for hospital at home.”  S2: “In Scotland we have services that use telemonitoring, but we don’t have well developed services for hospital at home. And it really got us thinking around that is our next priority area. […] I think if we haven’t come on this visit we might not have reached that point. So it really helped my thinking forward.” | Norrbotten indicated in the action plan that their “ambition is to design a common system for advanced care planning where patients can take an active role and communicate with healthcare professionals as required. Furthermore, they described that current systems need to be changed and redesign in order to offer patients the possibility to receive a safe and coordinated healthcare, across the organisational boundaries and where the patients are seen as an obvious part in planning of care process that concern them. Social care, health care and rehabilitation models need to be changed to accommodate citizens' needs and wishes.” | In the action plan of Puglia, the challenge was indicated as “health and social care seem to be often fragmented, with services based on professional and institutional boundaries rather than being co-ordinated around the needs of citizens. A number of policy initiatives in Puglia have been designed to tackle this fragmentation, however integration of health and social care still remains a challenge. And that one of the three main barriers to integration is lack of engagement of the “Third Sector” in participating in the delivery of integrated care services.”  A respondent provided a statement reviewing the problem:  P1: “From my point of view, we already knew something about the general framework and legislation in Scotland because we have been working quite a lot together. And we wanted just the confirmation whether legislation for integration is important and whether it can make a difference, and I now have the confirmation that it would make a difference in Puglia.” | For Norrbotten the challenge remaining as described in the action plan was “how to address a gap between emerging innovations and its implementation, monitoring and evaluations. As such, there is a real opportunity to learn from other regions and organisations in Europe how to manage innovation processes better and more effectively**.”**  Two of the respondents from Norrbotten indicated the following:  R2: “In terms of innovation management I think there are certain things were I see you come further, were we really want to work and improve and that is not that cohesive, much more of a cohesive process in Scotland in terms of innovation management. Mandate, clear mandates and roles, such an important aspects, of course funding is also important but just to have that, in Scotland you have 8 innovation centres with different responsibilities, in Norrbotten we do have different innovation centre, one joint with municipality and university. We all invested money into it, so were plouwing money into it but have a very unclear mandate. Very unclear.” | The challenge for the receiving region Olomouc as written in the action plan is that: “the role of ICT as an enabler of service redesign is not well recognised and digital healthcare services are not developed. Some progress has been made though for example by preparing a new Electronic Health Act, concept of sharing of health data and eHealth strategy (2016). […] The major issue for the Olomouc Region is to recognise the widespread of eHealth services as a routine part of the healthcare delivery and patient journey, wider Czech Republic the progress in this area has been made mostly on the voluntary basis of involved stakeholders. There is no ICT infrastructure to allow electronic exchange of data, including access to electronic health records. In addition, legislation is lacking to support a wider implementation of eHealth services in the Region.”  One of the respondents from Olomouc indicated the following:  CR2: “Importance of shared electronic health record and associated organisation measures, which I can explain. These are the aspects how to actually introduce the electronic health records, the shared electronic health record in environment that we have. That means with many health care providers different owners and also with their diverse information systems. And will need really more stronger reflection in either legislation in the new eHealth law, which is to be prepared soon. Or in other legislative documents. Because without it would be really difficult to build any integration care or to do integration care as such in health care. Because sharing the information is essential. It is one of the key points we learned here […]” |
| Receving region 2 | For Olomouc, the challenge was described in the fact that “the role of ICT as an enabler of service redesign is not well recognised and digital healthcare services are not developed.” […] Hence the opportunity to learn about these features and their potential transferability to Olomouc region.”  One respondent indicated about the problem:  C1: “This is not only the practice as such but also some idea for our environment, our context in the Czech Republic. What we can do as this quite critical to do the changes in our system which is quite rigid. Quite resistant to any kind of change, of that kind, that means integration of various levels, of integration of social care. That means that this was a very good experience here and also make use of all the opportunities to get more information how it is done.” |  | In the action plan of Basque country the problem was described as: “The Third Social Sector (TSS) in the Basque Country and the public sector collaborate in the provision of social services of general interest, however, the public sector needs to contemplate new forms of relationship with civil society that allow progress in a model of open administration and participatory governance and society.” […] “there is a need to involve the Third Social Sector in the provision of integrated care […], in particular in relation to financial sustainability of the services and lack of resources.”  One of the respondents from the Basque country indicated the following:  ES3: “From my point of view with the community at local level, this experience has helped me to realise that we shouldn’t make so much effort in trying to people participate in our initiatives. What we think is good for the community. Rather than watch and see where are they participating, where are they putting their interest, and try to facilitate their involvement in those activities.” |  |  |
