# Supplementary material for: The transfer of knowledge on integrated care among five European regions: a qualitative multi-method study
Source: BMC Health Serv Res. 2020 Jan 3;20:11. doi: 10.1186/s12913-019-4865-8 (PMC6942405; doi:10.1186/s12913-019-4865-8)
Supplement: Supplementary file 2 — Additional file 2. Classification of contextual dimensions (dimensions are described in the SCIROCCO tool) and organisational or professional contextual characteristics, type of knowledge mobilisers and receivers and type of knowledge transferred or needed during the knowledge transfer activities of SCIROCCO. [file 12913_2019_4865_MOESM2_ESM.docx]

| Additional file 2 Classification of contextual dimensions (dimensions are described in the SCIROCCO tool) and organisational or professional contextual characteristics, type of knowledge mobilisers and receivers and type of knowledge transferred or needed during the knowledge transfer (KT) activities of SCIROCCO. | | | |
| --- | --- | --- | --- |
|  | Transferring region | Receiving regions |  |
| Case 1 (Good practice in telemonitoring) | Puglia | Scotland | Olomouc |
| **Context** | |  |  |
| Not feasible to transfer |  | Information & eHealth, Finance & Funding |  |
| Feasible but with lots of efforts |  | -Removal of Inhibitors (Professional context: Recruitment of general practitioners (GPs) remains a big challenge […])  -Citizen Empowerment  -Evaluation Methods (Professional context: […] The resistance of healthcare professionals to share data needs to be better addressed as well.) | -Readiness to Change (Professional context: However, awareness raising activities may active a change and the need for change has already been recognised by many experts and managers actively participating in health and social care.)  - Structure & Governance, Information & eHealth (Organisational context: There are plans on the national level to develop solutions and platforms to allow information sharing. However, this is a very challenging issue due to fragmentation of care providers who own the information and communications technology (ICT) systems […])  -Standardisation & Simplification  - Finance & Funding  -Removal of Inhibitors (Professional context: There are essentially no recognised barriers on professionals’ side (managers, medical) to introduce innovations such as this good practice but the healthcare system is strongly based on fee-for-service scheme)  -Breadth of Ambition  -Innovation Management |
| Feasible with some/certain/further efforts |  | -Standardisation & Simplification -Population Approach | -Population Approach -Citizen Empowerment  -Evaluation Methods  -Capacity Building (Professional context: There is training of healthcare professionals in place, even though some Curricula should be upgrade) |
| Feasible with no need for major adaptation |  | -Readiness to Change -Structure & Governance -Breadth of Ambition -Innovation Management -Capacity Building |  |
| **Knowledge** (data retrieved from action plans, focus groups and study visit programmes) | |  |  |
| Type of knowledge donors (transferring region) and type of knowledge receivers (receiving regions)* | **Pra & Dev*** (P1: “We involved clinicians, nurses, technicians, also, company that implemented the technology”)  **DMs*** (P1: “[...] our policy maker that attended the meeting […]”), (“[…] I could see today from the directors that were really surprised and happy that we managed to get to the point to becoming interesting for other countries.)” | **Dev & DMs*** (S5: ”Well we approached people that we knew about telemonitoring from a policy and implementation perspective.”)(i.a. Service Development Manager, Head of International Engagement, International Engagement Manager Scottish Government TEC and Digital Health and Care Division) | **Pra*** (C1: “We took our clinicians together, that means to have a medical arm, to understand the visit from their point of view and it was very useful.”)  **Dev*** (Project Manager for International Projects) |
| Type of knowledge (offered by transferring region and needed by receiving region) (data retrieved from action plans, focus groups and study visit programmes)** | **Sc**** (The analysis of data related to the monitoring of about 100 patients allows better understanding of the effectiveness of the remote monitoring system and to what extent it improves the quality of care for patients. -There is an evidence that the good practice is economically viable, and it brings benefits to the target group.)  **T**** (S3: “I think the wealth of and generosity of sharing this information as you would say, the challenges, your whole experience, your generosity of sharing all of that amongst us all is very much appreciated.”)  **Wi**** (S2 about the practical site visit: “And that there were so many people there, the general practitioners, the consultants, nursing staff and technical staff, that showed that this was really a collaboration and we felt that.”) | Adaptation of the features in Scotland:  **Sc**** (Evaluation methods: Improve publishing of evaluation data and demonstrate the impact, Improve real-data collection)  **T**** (Citizen empowerment**:** Embed the education about the importance of citizen empowerment and active participation in schools’ curricula)  **Wi**** (Removal of inhibitors: Improve flexibility of engaging with GPs on the individual basis; there is no “one size fits all approach”, Encourage learning about the ways of how to engage with general practitioners and promote GPs nationally and beyond to facilitate scaling-up of successful stories), Citizen empowerment: (Increase public awareness about the benefits of technology enabled care solutions) | Adaptation of the features to Olomouc’s local context:  **T**** (Readiness to change: Introduce new legislation related to digital healthcare and integrated care; development of strategy of integrated care is currently on-going, Finance and Funding: Promote multi-sourced and coordinated funding which would include investment in technologies, updates of the reimbursement schemes […])  **Wi**** (Readiness to change: Organise information campaigns to raise awareness about the benefits of the good practice for the stakeholders involved, policy-makers and healthcare professionals in particular, Encourage new way of working; a need for improved collaboration and partnerships-building among stakeholders involved) |
| Case 2 (Good practice in advance care planning (ACP)) | Basque Country | Norrbotten |  |
| **Context** |  |  |  |
| Not feasible to transfer |  | Structure & Governance (Professional context: It would also need professionals who are motivated and have clear leadership in place who have knowledge of the benefits of more involved patients) -Finance & Funding -Removal of Inhibitors |  |
| Feasible but with a lot of efforts |  | Readiness to Change (Professional context: It is necessary to have a legal framework for integrated care solutions and ethics committees in place in order to ensure that new methods are in line with Norrbotten’s values and professional ethical codes for employees […])  -Standardisation & Simplification (Organisational context: The advanced care plan needs to be adapted in order to have homogeneous technical standards throughout the different organisations involved. The document needs to be accessible by patients and all healthcare professionals involved.) -Evaluation Methods -Capacity Building (Organisational context: The need for continuous learning needs to be embedded in the routine practice.) |  |
| Feasible with some/certain/further efforts |  | -Citizen Empowerment -Breadth of Ambition -Innovation Management |  |
| Feasible with no need for major adaptation |  | -Information & eHealth (Organisational context: There is an integrated infrastructure in place to allow sharing of clinical information between the different levels of care in Norrbotten. There is also integrated electronic health record in place.) -Population Approach |  |
| **Knowledge** |  |  |  |
| Knowledge donors/receivers* | **KPs*** (Research & Development Coordination Manager)  **Pra*** (Medical Doctor and Nurse)  **Dev***(Project Manager, Procurement and Insurance Directorate, Quality and Health Information System) **DMs*** (Head of Integrated care and Chronicity Service and Director) | **Dev*** (N6: “[…] she works with the development and very close to the clinical site, also much closer than we do, […].),  (Project Director of the Development department, Improvement Strategic Officer, Business developer)  **Pra* (**N6: “[…] she is a registered nurse and she works for a palliative care team”) |  |
| Type of knowledge** | **Sc**** (The good practice has not yet been formally evaluated. Nonetheless, taking into considerations the views and perceptions of participants in the ACP (patients, families, GPs and community nurses), it seems that the Practice has proven to be invaluable.)  **T**** (N4: “I think that expectations has been fulfilled, it was a really great opportunity to get here and to see in real life how it works and like this team plan today with the patient and doctors.”)  **Wi**** (N5: “And it is really interesting to hear also from the receiving region that like it is not the document itself, that is the most important. It is the process of talking with your family about these issues.”) | Adaptation of the features to Norrbotten’s context  **T**** (Readiness to change: Develop Implementation Plan for the adoption of good practice, Support the need for a change with empowering of healthcare professionals to implement ACP good practice through training and education. Information & eHealth Services: There is a need for a development of new Health and Social Care Plan and the documentation system […]) **Wi**** (Readiness to change: Raise awareness about the benefits of the good practice approach to good practice leaders and implementers.) |  |
| Case 3 (Dimension of the SCIROCCO tool: Innovation Management) | Scotland | Basque Country | Puglia |
| **Context** |  |  |  |
| Not feasible to transfer |  | Finance & Funding |  |
| Feasible but with lots of efforts |  | -Population Approach -Citizen Empowerment -Evaluation Methods  -Innovation Management  -Capacity Building | -Readiness to Change  -Structure & Governance -Information & eHealth -Standardisation & Simplification |
| Feasible with some/certain/further efforts |  | -Readiness to Change  -Structure & Governance  -Standardisation & Simplification | -Removal of Inhibitors (Professional context: There is a need for continuous training sessions for the stakeholders involved in the third sector. Information campaigns about the role and benefits of involving third sector in the care provision should be promoted)  -Population Approach  -Citizen Empowerment  -Evaluation Methods  -Innovation Management (Professional context: Continue with the training programmes for all stakeholders involved in the delivery of health and social care, including third sector.) |
| Feasible with no need for major adaptation |  | -Information & eHealth Removal of Inhibitors (Professional context: Some improvements need to be done around the implementation and change of culture), -Breadth of Ambition | -Finance & Funding, -Capacity Building (Organisational context: There is already an organisational structure in place to facilitate the training) |
| **Knowledge** |  |  |  |
| Knowledge donors/receivers* | **Pra*** (S1: “We primarily focused on the voluntary organisations. We tried to give the picture of the national versus what is happening at the local level or in practice.”)  **DMs*** (i.a. Policy and Development Officer, Coalition of Care and Support Providers)  **Dev*** (i.a. Service Development Manager)  **SUs*** (Site visits to two good practices) | **Dev & DMs*** (ES1: “What we tried to involve people from different specialities that hold the social and health system need. People from the social system, from the health system and the coordination of the health system. ES2:’’Concretely from community level, social level and the innovation level.”),  (i.a. Head of Integrated care and Chronicity Service and participants working for the Basque Government.)  **KPs*** (One participant worked at Basque Foundation for Health Innovation & Research) | **Dev & SUs*** (I1: “[…] we wanted to involve the innovation level which is the agency represented by us. And also health programming point of view, and the other hand is social programming view. And the civic presentation which is for us really important, their point of view.”)  **DMs*** (i.a. Social Programme Development Unit, Puglia Government.) |
| Type of knowledge | **Sci**** (In principle, the success of engagement of the third sector is measured by its contribution to National Health and Wellbeing Outcomes Framework)  **T**** (ES3: “And I am sure we can take bits from the site visit experience that we can take the most out of them within our environment and with our culture.”)  **Wi**** (I1: “Personally, I really liked that they involved us directly to transfer us the real meaning and the importance of what they do in the social service to inclusion. They made us play with iPad, it couldn’t be a better strategy because we had fun but at the same time we truly got the message.”) | Adaptation of features to the Basque Country’s context  **Sci**** (Evaluation Methods: […] Therefore, one option of how to improve evaluation of the third sector activities would be the creation of a working group […]. The objective of this group would be to identify a set of indicators to measure participation of the Third Social Sector (TSS) in the provision of integrated care which could be then included in the Osakidetza’s (regional public healthcare system) Framework Contract.)  **T**** (Structure and Governance: The objective of the Directorate is to: -develop integrative mechanisms between professionals in order to direct and coordinate the commission of health and social care in each Integrated Care Organisation.  -direct, promote and coordinate social and citizen participation in health and social care and thus enhancing citizens’ co-responsibility and self-management of their health. Innovation management**:** A possible action is to reinforce the Euskadi Lagunkoia initiative (aims to encourage the participation of older people and the general public to improve neighbourhoods and environments in the municipalities of Euskadi in order to continue living active life as we age.) in the three Basque provinces […]) | Adaptation of features to Puglia’s context  **T**** (Readiness to Change: Embed Third Sector reorganisation in the regional policies and planning.  Structure and Governance: Develop a roadmap for a change programme to unify social and health funding in order to deliver tailored solutions for chronic complex citizens.  Breadth of Ambition: Need to adapt and reform third sector legislation in order to remove an organisational and financial fragmentation and deliver full integrated services.)  **Wi**** (Readiness to Change: Foster voluntary workers involvement in institutional initiatives and in decision making in order to facilitate and favour cultural change.) |
| Case 4 (Good practice(s) in Third Sector) | Scotland | Norrbotten |  |
| **Context** |  |  |  |
| Not feasible to transfer |  | -Structure & Governance -Finance & Funding  -Removal of Inhibitors (Professional context: […] The dedication and continuous training of healthcare professionals seem to be crucial inhibitor in the Norrbotten’s local context.) |  |
| Feasible but with lots of efforts |  | -Readiness to Change -Evaluation Methods, -Capacity Building (Organisational context: The need for continuous learning needs to be embedded in the routine practice.) |  |
| Feasible with some/certain/further efforts |  | Standardisation & Simplification (Organisational context: The innovation management plan needs to be adapted in order to be compatible with technical standards of all organisations involved) -Citizen Empowerment -Innovation Management |  |
| Feasible with no need for major adaptation |  | -Information & eHealth,  -Population Approach -Breadth of Ambition (Organisational context: There is an integration between primary and hospital care levels established in the region.) |  |
| **Knowledge** |  |  |  |
| Knowledge donors/receivers* | **KPs*** (Head of Planning and Performance, Digital Health and Care Institute)  **DMs*** (i.a. Strategic partnership Director, Digital Health and Care Institute, Design Director, Digital Health and Care Institute, CTO Digital office.)  **Dev* (**i.a. International Engagement Manager,  Head of Planning and Performance, Digital Health and Care Institute) | **Dev*** (N2:”The three of us work for the development department, innovation is our responsibility. It is our area, to support research, innovation, improvement and eHealth development.”) ( i.a. Project Manager, Innovation Developer, Improvement Strategic Officer)  **Pra*** (N4: “I have been part of the working group of the SCIROCCO project, I ended up there because I was working with integrated care as a nurse and have experience in development, implementation and maintenance.”) |  |
| Type of knowledge ** | **T**** (presentations i.a. on Introduction to Digital Health and Care Institute (DHI), DHI Innovation model and methodology, Innovation management in Scotland, Involvement of regional health and social care authorities, universities and private sector companies and other sectors in the innovation process (i.e. “open innovation’’) and creating the culture of change) | **T**** (Readiness to Change: Develop implementation plan for the adoption of innovation, endorsed by the policymakers, including the clear assignments of roles and leaderships of all stakeholders involved in the implementation of new innovation processes. Innovation management: Develop instruments to support innovations.)  **Wi**** (Innovation management: Raise awareness about the need for innovation and new way of working. Readiness to Change: Raise awareness about the benefits of innovations to leaders and implementers.) |  |
| Case 5 (Dimension of SCIROCCO tool: Information and eHealth) | Norrbotten | Olomouc |  |
| **Context** |  |  |  |
| Not feasible to transfer |  |  |  |
| Feasible but with lots of efforts |  | -Readiness to Change (Professional context: […] However, awareness raising activities may active a change and the need for change has already been recognised by many experts and managers actively participating in health and social care.)  -Structure & Governance (Professional context: The first step to be introduced can be around the awareness raising about the need and benefits of eHealth services)  -Information & eHealth -Standardisation & Simplification  -Finance & Funding -Removal of Inhibitors  -Breadth of Ambition  -Innovation Management |  |
| Feasible with some/certain/further efforts |  | -Population Approach -Citizen Empowerment  -Evaluation Methods  -Capacity Building (Professional context: There is training of healthcare professionals in place, even though some Curricula should be upgrade. There is a lot of ad hoc education at the pilot phase which should be expanded to continuous learning and training) |  |
| Feasible with no need for major adaptation |  |  |  |
| **Knowledge** |  |  |  |
| Knowledge donors/receivers* | **KPs*** (CEO,Luleå University of Technology.)  **DMs** *(i.a. Deputy Regional Director) **Dev*** (E-health Strategist)  **Pra*** (Chief physician, surgery, Registered Nurse and Head of a primary care unit) | **Pra* (**Medical doctor) **Dev*** (i.a. Project Manager for International Projects.)  **DMs*** (Head of Heart Failure Department) |  |
| Type of knowledge** | **T****(i.a. presentations on Introduction to EHealth in Region Norrbotten, Introduction to EHealth in Region Norrbotten,  Infrastructure for sharing health data between various healthcare providers, Qualifications of personnel in telemedicine services.)  **Wi**** (CR4: “I came here to get some practical information for telemedicine for especially heart failure patients, so were looking forward today to see the real practice in hospital. So it was a little bit, I haven’t seen so much as I expected, but what I appreciate is how the system is done here in Sweden, I liked the electronic health record and the information from the patients is available to everyone, this was very…”) | Adaptation of features to Olomouc’ local context  **T**** (eHealth services: -Develop mechanisms to reduce the complexity of introducing the concept of eHealth services. The ICT solutions to allow information sharing between various healthcare providers are expected to be soon developed. -Develop mechanisms to improve the communication and collaboration of key stakeholders by creating a joint committee between Ministry of Labour and Social Affairs and the Ministry of Health in order to better coordinate implementation of ICT solutions and raise awareness about the need to extend the sharing of health data to social care providers. This is currently not envisaged in eHealth strategy. -Develop mechanisms to enhance citizens empowerment and proactive approach of citizens to manage their own health and self-care. Changes in the reimbursement system and payment schemes can be seen as one of the incentives.)  **Wi**** (eHealth services Raise awareness and promote the benefits of eHealth services in order to speed up the implementation of new national eHealth strategy.) |  |

*****Categories retrieved from Ward (et al) [6,9]: Categories for type of knowledge donor/receiver: Professional knowledge producers who produce empirical and/or theoretical knowledge and evidence (**KPs**), Frontline practitioners and service providers responsible for delivering services to members of the public (**Pra**), Members of the public acting as or on behalf of their communities and people in receipt of services (**SUs**), Decision makers responsible for commissioning services and/or designing local/ regional/national policies and strategies + policy makers (**DMs**), Product and programme developers responsible for designing, producing and/or implementing tangible products, services and programmes (**Dev**). ******Categories for the type of knowledge: Scientific / factual knowledge – research findings, quality and performance data, population data and statistics, evaluation data (**Sc**), Technical knowledge – practical skills, experiences and expertise (**T**), Practical wisdom – professional judgments, values, beliefs (**Wi**).
