# Supplementary material for: The transfer of knowledge on integrated care among five European regions: a qualitative multi-method study
Source: BMC Health Serv Res. 2020 Jan 3;20:11. doi: 10.1186/s12913-019-4865-8 (PMC6942405; doi:10.1186/s12913-019-4865-8)
Supplement: Supplementary file 3 — Additional file 3. Type of Intervention, including responsible actors and Use of knowledge as indicated by the receiving regions. [file 12913_2019_4865_MOESM3_ESM.docx]

Additional file 3 Type of Intervention, including responsible actors and Use of knowledge as indicated by the receiving regions.

| **Intervention** | Receiving regions |  |
| --- | --- | --- |
| Case 1 (Good practice in telemonitoring) | Scotland | Olomouc |
| I**ntervention** |  |  |
| Clarifying the type of intervention to be used (information management,  linkage, decision/  implementation support,  capacity development) | **Capacity development** **and Linkage**  Engagement with good practices: Objective of this action is to increase the engagement of general practitioners in the delivery of technology enabled care solutions, promoting the real benefits and opportunities of these solutions.  Public awareness and engagement of citizens in service redesign: Objective of this action is to organise and promote awareness-raising sessions about the benefits of active engagement of citizens in the service redesign. This includes engagement with educational sector and embedment of the citizen empowerment in teaching’s curricula.  **Information management**  Systematic evaluation and data collection: The objective of this action is to support publishing of evaluation data in the right time in order to demonstrate the value and impact of technology enabled care services. This also includes the quality of data collection in the real-life settings and better use of data collection infrastructure.S1: “Well I think we spoke quite a bit about data and data collection and what the opportunities might be to improve that because it plays such a key role.” | **Capacity development** **and Linkage** Awareness raising campaigns: -Increase awareness about the need for integrated care and its benefits for stakeholders involved.  -Create conditions to support a change towards more connected and coordinated health and social care services.  -Influence the planning and design of integrated care concept.  -Create a shared vision for integrated care. -Raise awareness about a new way of working; partnership-building approach and its benefits.  **Capacity development** **and Decision and implementation support** Create a shared vision for integrated care:  Use of European Regional Development Fund (ERDF) to develop and implement a complex strategy for integrated care and digital healthcare.  **Information management**  C1: “When we were sitting here, and doing the exercise with the scirocco tool we predesigned a good practice. […] good basis for a new project that would integrate care of the cardiologists, that are not within the hospitals and are somewhere in the regions, and also the general practitioners that take care of the particular patient, how was in care of our hospital. That means to extent the information about the care that was given to the patient in the hospital also to specialist, cardiologist for example and general practitioner, which is associated with the patients.” |
| Responsible actors | A total of 9 responsible actors were listed including e.g. the government, partnerships and National Health Service (NHS). | No specific responsible actors were indicated. C1: “[…] We are preparing proposals for this including technical specification and other necessary documents that would specify in price, the equipment, we know how to do it and we know we can estimate that it will be done in outline. We have direct access to decision-makers.” |
| **Use** |  |  |
| Deciding how the  knowledge will be used: knowledge was used in a range of different ways: directly (i.e. with little modification), conceptually (i.e. to change opinions) or politically (i.e. to confirm or challenge practices or policies) (Weiss,1979). | Knowledge will be used conceptually | Knowledge will be used conceptually and politically |
| Knowledge used politically | Policy implications: a total of four existing programmes, plans and strategies were described which support the priority actions in Scotland. | There has been a new strategy developed for integrated care at national level in Czech Republic in August 2018. New models of care are introduced and piloted across the country. As such, there is an opportunity to feed the learning about Hospital@Home good practice rather than reinvent the wheel. |
| Practicalities of use / why knowledge is being mobilised?  • To develop local solutions to practice-based problems (So)  • To develop new policies, programmes and/or recommendations (Po)  • To adopt / implement clearly defined practices and policies (Imp)  • To change practices and behaviours (Ch)  • To produce useful research / scientific knowledge (Kno) | • To (further) develop new policies, programmes and/or recommendations **(Po);**  •To change practices and behaviours **(Ch):**  The learning about the Hospital@Home good practice showed clear similarities of Puglia Region and Scotland’s vision of how to transform care delivery. The main focus in both regions is to look for the primary care led solutions which would help to shift the balance of care from hospital to community settings, increase capacity and reduce the demands on health and social care services. A number of opportunities were identified in Scotland, namely:  -Opportunity to improve engagement of general practitioners (GPs) in the delivery of technology enabled care solutions. […] -Opportunity to improve the funding of care transformation. […] -Opportunity to better promote benefits and impact of technology enabled care solutions in order to facilitate their ’buying.’ […] -Opportunity to better target citizens who would benefit most from technology enabled care solutions. […] | • To develop local solutions to practice-based problems **(So);**  • To (further) develop new policies, programmes and/or recommendations **(Po);**  •To adopt / implement ideas of transferring regions on practices and policies **(Imp)**:  […] would contribute to the improvement of a number of outcomes:  -Decreased a need for hospital beds  -Improved care of chronically ill, including those discharged from hospital  -Reduced number of reduce the number of unstable patients with chronic diseases. |
| Case 2 (Good practice in advance care planning) | Norrbotten |  |
| Clarifying the type of intervention to be used | **Information management**R5: “I think I reflected with the other participants yesterday about this as she is not here today. We said that one thing we take with us, but that is maybe not doable right now, is that the timeline which you had in your journal (sort of electronic care plan) that also contained information from other care givers as well, that was like a really wauw we would love to have it.”  **Decision and implementation support and Linkage**  Develop an implementation plan for the advanced care planning (ACP) good practice:  Introduce new way of working that extends the current organisational barriers, including responsible actors, leadership, processes and anticipated duration.  Introduce a new Health and Social Care Plan:  Improve citizen empowerment and engagement in the decision-making processes in the planning and implementation of health and social care interventions.  **Capacity development and Linkage**  Improve education of healthcare professionals:  Join the efforts in providing the same level of education and training to all healthcare professionals involved. |  |
| Responsible actors | Not indicated in action plan, but in focus groups.: R6 indicated: “Expect to our daily work we have a local steering group, which has some of the decision-makers which are also responsible for regional wide decision. They have the responsibility, from our steering group, to bring that further. So we report to them, we give them suggestions, and we say these are the actions we need to take. And this will also be part, as we report to them, what we do in the project. […] From this best practice, this is also, in terms of the analyses and what we should do from now on, we also recommend these steps within this area, and then they are responsible for actually handling these results.” |  |
| Deciding how the  knowledge will be used: knowledge was used in a range of different ways - directly (i.e. with little modification), conceptually (i.e. to change opinions) or politically (i.e. to confirm or challenge practices or policies) (Weiss,1979). | Knowledge will be used directly, conceptually (i.e. to change opinions) and politically |  |
| Knowledge used politically | In order to implement the ACP good practice the following policy actions need to be considered:  -the good practice needs to be embedded in the existing policies and strategies related to integrated care and digital healthcare in order to secure the leadership for its implementation. The new way of working could be well integrated into the operating care model in Norrbotten Region and as a part of new Strategy for Future Health Care.  -Adaptation of the Advanced Care Plan to comply with the technical standards across the different organisations. New guidelines and standards are required for the entire nation, to facilitate regional decisions on changing methods.  -Adaptation of the funding system to support the time release of healthcare professionals. Current system is based primarily on the number of visits needs to change to calculate the value for the patient. This is a system shift that takes time in a hierarchical organisation. […] |  |
| Practicalities of use / why knowledge is being mobilised?  • To develop local solutions to practice-based problems (So)  • To develop new policies, programmes and/or recommendations (Po)  • To adopt / implement clearly defined practices and policies (Imp)  • To change practices and behaviours (Ch)  • To produce useful research / scientific knowledge (Kno) | •To change practices and behaviours **(Ch)**;  •To (further) develop new policies, programmes and/or recommendations **(Po);**  •To adopt / implement ideas of transferring regions on practices and policies **(Imp):**  The adoption of ACP good practice in Norrbotten Region would enhance a new way of working in planning and implementation of health and social care interventions for patients in a need for advanced care planning. It would improve the opportunities for the patients to make their own decisions on care, including end-of-life care. This approach would complement new Strategy of Norrbotten Region: The Road to the Future Health and Care which outlines a paradigm shift for healthcare from citizens” perspective. The approach will change working methods and create new services that shape a new care delivery; a healthcare that meets the needs of each person on equal basis. The new ways of working will also facilitate the provision of new skills required for such a change. |  |
| Case 3 | Basque Country | Puglia |
| Clarifying the type of intervention to be used  These included information management  (e.g. gathering, sharing and packaging information), linkage  (e.g. bringing people together or facilitating dialogue), capacity  development (e.g. learning from the KE process  and ensuring sustainability) and decision and implementation  support (e.g. advising as a critical friend/outsider). | **Capacity development and Linkage**  Strengthen the representation of the third sector in various participation bodies at Integrated Care Organisations (ICOs)’ level: Encourage the third sector to participate in decision making processes, regarding population’s care in the area where the ICOs operate.  **Information management and Linkage**  Include indicators on participation of third sector in the provision of integrated care into the Osakidetza”s Framework Contract and the Preferred Offer of ICOs:  Promote communication between the ICOs and the third sector, agree common objectives and involve the third sector as an active agent in the provision of integrated care.  ES3: “And I am sure we can take bits from the good practice site visit experience that we can take the most out of them within our environment and with our culture. And also, we have to see how the structure, the governmental structure as department of health, department of social and research innovation, how all the blocks are put together to make it easy for implementation in the local level.”  **Decision and implementationsupport and Linkage**  Reinforce the Euskadi Lagunkoia initiative in the three Basque provinces and extend Adinberri Gipuzkoa to the whole Basque Country: To extend an innovative initiative that encourages the participation of all the actors involved in the care continuum of older people, promotes and environment of cooperation towards the common objectives. | **Decision and implementation support**  Reform of the third sector at a regional level: Embed third sector collaboration in the regulation and policies related to health and social care service delivery.  **Information management, Capacity development and Linkage**  Reform of the third sector at a regional level: Map and coordinate third sector initiatives including at a regional level and thus facilitate the partnership building in order to systematically share strategies and co-design the Action Plans. **Capacity development and** **Decision and implementation**  Integration of funding system: Overcome the fragmentation of funding for integrated care service  Promote the scaling up of existing pilots (e.g. Buoni Servizio) carried out in Puglia on the definition of “Health and Social Care Pathways” and related co-payment system “concept” to be shared between health and social sector (integration of funds)  **Information management and Capacity development**  Improved data collection and information sharing  Make possible the full implementation of the concept of personalise medicine and “big data” in order to inform the definition of the Health and Social Care Pathways and protocols.  Accelerate the integration of information and communications technology (ICT) platform in order to share data (across health and social care settings)  **Information management and Linkage**  I1: “Yes, certainly this self-directed support is something I will bring back home and discuss with my directors.” |
| Responsible actors | Department of Employment and Social Policies, Foral Deputations of the three provinces, Department of Health, Osakidetza’s (regional public health systems) Executive. | The regional Agency for Health and Social Service,Department for Health Promotion, Social Affair and Sports for all. |
| Deciding how the  knowledge will be used: knowledge was used in a range of different ways - directly (i.e. with little modification), conceptually (i.e. to change opinions) or politically (i.e. to confirm or challenge practices or policies) (Weiss,1979). | Knowledge will be used conceptually and politically | Knowledge will be used conceptually and politically |
| Knowledge use politically | Policy implications:  -It will be necessary to extend health strategic lines of both the Department of Health and Osakidetza’s Health Plan, to involve third sector’s representatives in the Osakidetza’s ICOs.  -It will be necessary to reinforce transversal evaluation to fortify horizontal integrated care and strengthen the coordination between stakeholders involved.  -It will be necessary to extend innovation in the health sector to include the Third Social Sector (TSS) organisations. | -The regional Agency for Health and Social Service provides the technical support for Department for Health Promotion, Social Affair and Sports for all.  -The Agency main role is to foster health and social Innovation processes in the region. As such, the Agency will be involved in developing these priority actions further, e.g. by forecasting the skills, competences and knowledge needed for their implementation, including the development of feasibility study and SWOT analysis. As a result, the Agency might consider useful to propose to the Department for Health Promotion, Social Affair and Sports for all to develop a Memorandum of Understanding with Scotland as a coaching region in order to support the transferability, adaptation and embedment of this successful experience of Scotland in engaging the third sector in the provision of integrated care. |
| Practicalities of use / why knowledge is being mobilised?  • To develop local solutions to practice-based problems (So)  • To develop new policies, programmes and/or recommendations (Po)  • To adopt / implement clearly ~~defined~~ practices and policies (Imp)  • To change practices and behaviours (Ch)  • To produce useful research / scientific knowledge (Kno) | • To develop new policies, programmes and/or recommendations **(Po);**  • To change practices and behaviours **(Ch):**  There is a clear need to create a framework that defines the relationship between the health, social and third sectors in the Basque Country, including:  -Agreement on common objectives and creating a vision of “working together”  -Prioritisation of activities  -Involvement of sectors in the decision-making bodies related to integrated care  -Identification of added value of “working together” approach and breaking down the silos  -Promotion of intersectoral communication and collaborations  -Promotion of citizen participation in health matters.  Involving the third sector in the provision of integrated care would guarantee the most appropriate response to the needs of citizens at a right time; providing the citizens with resources and capacity to make and act upon their own decisions.  In addition, involving the third sector in the provision of integrated care would also mean greater efficiency and use of resources and capacities in the Basques society.  The Third Social Sector benefits from a better knowledge of people needs due to its proximity, empathy and active listening of citizens and promoting their active participation in the society. In turn, this would mean bringing citizens closer to the administration which would potentially lead to an improvement in the provision of coordinated and integrated care. | • To develop new policies, programmes and/or recommendations **(Po);**  • To change practices and behaviours **(Ch);**  • To adopt / implement ideas of transferring regions on practices and policies **(Imp):**  In Puglia, there are several third sector organisations (TSOs), however, their activities seem to be rather fragmented and not strongly aligned with a common integrated care vision. Scotland’s experience can help the Puglia Region to systematise the activities of the third sector by creating a more homogeneous regulatory and organisational framework in order to improve the involvement of TSOs.  In Scotland there are many TSOs working in social care, providing support for vulnerable and marginalised groups who frequently face poverty, social care needs and poor health. There are also organisations working in prevention, particularly in the area of food and healthy eating initiatives. Working closely with communities is a key remit of the third sector approach. In many case, organisational structure and aims are defined by the needs of a particular community in order to fill the gaps in the service provision. Third sector initiatives are very often are based on the development of social networks which are very powerful tool to improve social capital and reduce isolation. This in turn results in the improvement of health outcomes.  There is now a consensus that health and social care services in Puglia Region need urgent attention. Knowledge and ability to respond to this need is often hampered due to complicated communication channels. As organisations embedded in service users’ communities, TSOs are often able to overcome these communication barriers. This Action Plan will aim to demonstrate that TSOs have the potential to meet the growing needs and positively contribute to the improvement of integrated care services in Puglia region. |
| Case 4 | Norrbotten |  |
| Clarifying the type of intervention to be used  These included information management  (e.g. gathering, sharing and packaging information), linkage  (e.g. bringing people together or facilitating dialogue), capacity  development (e.g. learning from the KE process  and ensuring sustainability) and decision and implementation  support (e.g. advising as a critical friend/outsider). | **Decision and implementation support** **and Linkage**  Develop an implementation plan for innovation management: Stimulate innovations that extend the current organisational barriers, including organisation of responsible actors, leadership, processes and anticipated duration.  **Capacity development and Linkage**  Improve education of leaders: Join the efforts in providing the same level of education to all leaders involved in innovation management.  Visualise good examples of innovation to workforce and wider citizens: Improve citizen empowerment and engagement in the decision-making processes in the planning and implementation of health and social care interventions.  R2: “In terms of innovation management I think there are certain things were I see you come further, were we really want to work and improve and that is not that cohesive, much more of a cohesive process in Scotland in terms of innovation management. Mandate, clear mandates and roles, such an important aspects.” |  |
| Responsible actors | Not mentioned, but in focus group R2 mentioned: “I think both, within the project and outside, because we have a steering group that is responsible for the regional results. They are also responsible for handling the suggestions, actions we suggest. Beside of that our development department we have the role and responsibility of supporting innovation management. There are things we can already point out to our director of development what see needs to bring forth to the table to take decisions on.” |  |
| Deciding how the  knowledge will be used: knowledge was used in a range of different ways - directly (i.e. with little modification), conceptually (i.e. to change opinions) or politically (i.e. to confirm or challenge practices or policies) (Weiss,1979 | Knowledge will be used conceptually and politically |  |
| Knowledge use politically | In order to improve innovation management, the following policy actions need to be considered:  -Guidelines and a strategy for innovation management needs to be embedded in the existing policies and strategies related to development of methods and technologies in order to secure the leadership for implementation. The innovation management could be integrated as a part of new Strategy for Future Health Care in Region Norrbotten.  -Adaptation of the innovation management at a regional level. New guidelines and standards are required for the entire nation, to facilitate regional decisions on implementation of innovation management.  Strategies and guidelines for training on the use of innovative methods need to be developed and implemented in e-learning platforms.  -Documents and policies that statutes how the dissemination of innovative solutions need to be visualised. |  |
| Practicalities of use / why knowledge is being mobilised?  • To develop local solutions to practice-based problems (So)  • To develop new policies, programmes and/or recommendations (Po)  • To adopt / implement clearly defined practices and policies (Imp)  • To change practices and behaviours (Ch)  • To produce useful research / scientific knowledge (Kno) | • To change practices and behaviours **(Ch);**  • To develop new policies, programmes and/or recommendations **(Po):**  […] The need for innovation and innovative solution of working are greatly recognised as a priority for the Norrbotten’s healthcare system. The region is very much mature in innovation and its spread is encouraged at every level of health and social care. However, the overall strategy and plan how to manage the innovation and scale-up innovative solutions on large scale remains a challenge. Improved innovation management can lead to a number of benefits for Norrbotten’s healthcare system including:  -Improved access to care that is tailored to the individual needs of citizens;  -Improved efficiency of working methods and workforce organisation;  -Improved cost-effectiveness and cost-efficiency of health and social care. |  |
| Case 5 |  |  |
|  | Olomouc |  |
| Clarifying the type of intervention to be used  These included information management  (e.g. gathering, sharing and packaging information), linkage  (e.g. bringing people together or facilitating dialogue), capacity  development (e.g. learning from the KE process  and ensuring sustainability) and decision and implementation  support (e.g. advising as a critical friend/outsider). | **Capacity development** Improved awareness and recognition of the need for eHealth services: The objective of this action is to increase awareness of the key stakeholders of the benefits of eHealth services in order to speed up the adoption of new eHealth strategy.  Inform about new technology enabled care services: The objective of this action is to raise awareness about new technology enabled care services and their benefits, e.g. video-conferencing system. Position the role of the University Hospital Olomouc (UHO): The objective of this action is to raise the profile of the UHO in developing ICT infrastructure for information sharing.  CR3: “The shared documentation I think it can be a great benefit for all the doctors, maybe the communication with patients, we do conference, is something which is not widely accepted in our country. But maybe for some kind of patients it could be real advantage not to go to hospital because of age and comorbidities and so on. For sure, there was a number of situation or points which are useful which are inspiration for the modification in our approaches for our country.” |  |
| Responsible actors | […] the coordination and clear definition of responsibilities of various stakeholders involved in the implementation process need to be addressed in order to manage this change effectively. This in particular involves the collaboration of four key stakeholders: Ministry of Health, insurance companies, healthcare providers and medical societies. |  |
| Deciding how the  knowledge will be used: knowledge was used in a range of different ways - directly (i.e. with little modification), conceptually (i.e. to change opinions) or politically (i.e. to confirm or challenge practices or policies) (Weiss,1979 | Knowledge will be used conceptually and politically |  |
| Knowledge use politically | Positioning of eHealth agenda in Olomouc Region and wider Czech Republic is a very complex and long process. The main issue remains political sensitiveness of this agenda which strongly affects the allocation of budget and planned investments in this area. […]  However, the introduction of new eHealth strategy in 2016 as a legal framework for the implementation of ICT solutions may help to address this issue. The University Hospital Olomouc plays a very active role in contributing to the implementation of strategy and is one of the key players that can help to implement the priority actions defined in this plan. |  |
| Practicalities of use / why knowledge is being mobilised?  • To develop local solutions to practice-based problems (So)  • To develop new policies, programmes and/or recommendations (Po)  • To adopt / implement ideas and policies (Imp)  • To change practices and behaviours (Ch)  • To produce useful research / scientific knowledge (Kno) | • To change practices and behaviours **(Ch);**  • To develop new policies, programmes and/or recommendations **(Po);**  •To adopt / implement ideas of transferring regions on practices and policies **(Imp):**  A number of opportunities for improvement of eHealth services in Olomouc Region and the Czech Republic were identified at both strategic and implementation level.  Electronic exchange of health information between a variety of healthcare providers is an inherent part of implementation of eHealth services in Norrbotten Region as well as across Sweden. As the implementation of this concept in Olomouc Region does not progress sufficiently, compared to other European Union countries, further efforts will need to be made around the promotion of benefits of using eHealth services as part of the routine operation of all healthcare providers. As a result, both the healthcare system as well as patients will benefit from this opportunity in terms of accessing the accurate health data in the right time and right place.  The study visit to Norrbotten Region inspired the visiting clinicians which suggested to promote and inherit this concept of data exchange in order to improve current workflow. This new concept should be tested in Moravia in Olomouc Region for the patients with advanced heart failure. In addition, the University Hospital in Olomouc Region is planning to upgrade its ICT system and introduce a concept of data exchange. Outcomes of the twinning activities with Norrbotten Region will directly inform these developments. |  |
